# An Emerging Role for the Unfolded Protein Response in Pancreatic Cancer

**DOI:** 10.3390/cancers13020261

**Published:** 2021-01-12

**Authors:** Claire M. Robinson, Aaron Talty, Susan E. Logue, Katarzyna Mnich, Adrienne M. Gorman, Afshin Samali

**Affiliations:** 1 Apoptosis Research Centre, School of Natural Sciences, National University of Ireland, H91 W2TY Galway, Ireland; claire.robinson@nuigalway.ie (C.M.R.); a.talty2@nuigalway.ie (A.T.); katarzyna.mnich@nuigalway.ie (K.M.); adrienne.gorman@nuigalway.ie (A.M.G.); 2 Department of Human Anatomy and Cell Science, Rady Faculty of Health Sciences, Max Rady College of Medicine, University of Manitoba, Winnipeg, MB R3E 0J9, Canada; susan.logue@umanitoba.ca; 3 Research Institute in Oncology and Hematology, Cancer Care Manitoba, Winnipeg, MB R3E 0V9, Canada

**Keywords:** activating transcription factor 6 (ATF6), endoplasmic reticulum (ER), inositol-requiring enzyme 1 (IRE1), protein kinase RNA-like ER kinase (PERK), unfolded protein response (UPR)

## Abstract

**Simple Summary:**

Pancreatic cancer refers to a group of malignancies of which pancreatic ductal adenocarcinoma (PDAC) is the most common form. It is an aggressive tumour with few treatment options and very poor outcomes. There is an unmet need for novel targeted therapies for PDAC. In this regard, a better understanding of PDAC biology and in particular new pathways that contribute to disease progression would help identify novel targets for therapeutic intervention. Growing evidence implicates the unfolded protein response (UPR) in many cancers, and this article explores the evidence that supports a role for the UPR in PDAC.

**Abstract:**

Pancreatic ductal adenocarcinoma (PDAC) is the most common form of pancreatic cancer and one of the leading causes of cancer-associated deaths in the world. It is characterised by dismal response rates to conventional therapies. A major challenge in treatment strategies for PDAC is the presence of a dense stroma that surrounds the tumour cells, shielding them from treatment. This unique tumour microenvironment is fuelled by paracrine signalling between pancreatic cancer cells and supporting stromal cell types including the pancreatic stellate cells (PSC). While our molecular understanding of PDAC is improving, there remains a vital need to develop effective, targeted treatments. The unfolded protein response (UPR) is an elaborate signalling network that governs the cellular response to perturbed protein homeostasis in the endoplasmic reticulum (ER) lumen. There is growing evidence that the UPR is constitutively active in PDAC and may contribute to the disease progression and the acquisition of resistance to therapy. Given the importance of the tumour microenvironment and cytokine signalling in PDAC, and an emerging role for the UPR in shaping the tumour microenvironment and in the regulation of cytokines in other cancer types, this review explores the importance of the UPR in PDAC biology and its potential as a therapeutic target in this disease.

## 1. Pancreatic Cancer

Pancreatic cancer refers to a group of malignancies that originate from the pancreatic tissue. The majority of these malignancies arise from the glandular exocrine tissue, potentially from both ductal or acinar cells, and are referred to as pancreatic ductal adenocarcinoma (PDAC). PDAC is by far the most common form of pancreatic cancer, representing more than 90% of all pancreatic cancer cases [1]. Although the incidence of PDAC is relatively low, accounting for only 2–3% of all cancers, the mortality rate is remarkably high and is the fourth most frequent cause of cancer-related deaths worldwide [1]. PDAC has a five-year survival rate of less than 8%, due to the rapid development of advanced disease or metastasis [1].

Conventional treatments such as chemotherapy, surgery and radiation do not significantly improve survival rates for PDAC patients. Although resection and adjuvant chemotherapy is the only potential curative treatment approach, only 10–20% of patients present with resectable PDAC, and the five-year survival rate for these patients is only 15–25% [2,3]. The majority of patients (80–90%) present with non-resectable, locally advanced or metastatic disease at the time of diagnosis [2,3]. The overall survival for metastatic pancreatic cancer remains poor, and less than 20% of patients survive past the end of the first year [4].

Historically, the standard-of-care chemotherapy for PDAC was gemcitabine. However, first line therapies now tend to be FOLFIRINOX (a combination of oxaliplatin, irinotecan, 5-fluorouracil and leucovorin) or a combination of gemcitabine plus nanoparticle albumin-bound (nab)-paclitaxel [5]. Although chemotherapeutic intervention can improve survival of patients with early-stage pancreatic cancer, these treatments grant limited benefits to patients with late stages of the disease who have short life expectancies [6].

While the molecular pathways involved in the development, progression and metastasis of PDAC are not fully elucidated, mutations in genes such as *KRAS*, *CDKN2A/p16*, *TP53* and *SMAD4* and activation of their associated downstream signalling pathways appear to play a key role in the disease [7]. KRAS mutations are the most frequent mutations in PDAC, present in 90–95% of tumours [8]. These mutations arise early in disease and promote tumorigenesis [9]. While oncogenic KRAS was traditionally thought to be undruggable, recent advances suggest otherwise. Small molecule inhibitors that directly target KRAS^G12C^, as well as the development of inhibitors of Src homology-2 domain-containing protein tyrosine phosphatase-2 (SHP2), a non-receptor protein tyrosine phosphatase that modulates RAS activity [10,11], have yielded promising pre-clinical results, although their clinical benefits remain to be determined [12].

The poor prognosis associated with PDAC is largely due to both intrinsic and acquired chemoresistance. Components of the tumour microenvironment (TME), also known as the tumour stroma, and associated desmoplasia are major contributing factors to chemoresistance in PDAC. Desmoplasia, a key pathological characteristic of PDAC, occurs as a result of a dramatic increase in the proliferation of alpha-smooth muscle actin-positive fibroblasts and is accompanied by increased deposition of extracellular matrix components (ECM). In this environment, extensive extracellular matrix deposition alongside rapidly dividing tumour cells that often outgrow vascular supply leads to oxygen and nutrient deprived regions of tumour tissue. These environmental stresses cause PDAC cells, as well as cells within the stromal tissue, to rely on adaptive mechanisms to survive and thrive.

The unfolded protein response (UPR), a well-characterised cellular stress response pathway, is activated in many cancers, due to several factors which may include oncogenic stress, metabolic reprogramming as well as the harsh conditions in the TME. The UPR is an area of intense investigation for therapeutic intervention in cancer. In this review article, we will describe PDAC and its unique TME/stroma and discuss the emerging roles of the UPR in PDAC progression and how the UPR may regulate PDAC-stromal crosstalk and contribute to chemoresistance.

## 2. Pancreatic Tumour Microenvironment (TME)

The TME can occupy up to 90% of the entire tumour mass and comprises both cellular and acellular components [5]. High stromal content in PDAC is a consequence of the activity of cancer associated fibroblasts (CAFs) resulting in increased deposition of extracellular matrix (ECM) components [13]. Other cellular components of the PDAC TME are immune cells, endothelial cells, pericytes and nerve fibers. A wide range of soluble factors including cytokines, chemokines and growth factors co-exist alongside the acellular extracellular matrix and comprise the molecular component of the TME. Under normal circumstances, these cells and factors act to maintain the health of the pancreatic tissue by providing architectural support and paracrine signalling that enable pancreatic cells to perform their physiological activities. Upon injury, including during tumorigenesis, the balance between the pancreatic cells and the tissue microenvironment is perturbed, and there is significant expansion of the stroma. Interestingly, PDAC-related research has focused on the role of pancreatic stellate cells (PSCs) in tumour progression, as these cells are proposed to be the main contributors to the extensive extracellular matrix deposition [14].

## 3. Pancreatic Stellate Cells (PSCs)

PSCs are the most prominent cell type in the PDAC stroma and can constitute about 50% of the total tumour mass. They are periacinar star-shaped cells that were first isolated from the pancreas in 1998 and characterised as having striking similarities to hepatic stellate cells (HSCs) [13,15]. PSCs in the pancreas exist in two states: quiescent (qPSC) and activated (aPSC). In general, qPSCs are considered to be “housekeeper” cells of healthy pancreatic tissue; however, their precise role(s) in healthy pancreata remains relatively ill-defined. qPSCs are characterised by vitamin A storage droplets that decorate their cytoplasm and are thought to be important in maintaining pancreatic tissue homeostasis.

Alcohol consumption, smoking, environmental stresses such as hypoxia, hyperfusion, oxidative stress and excessive cellular signalling by growth factors and/or cytokines can all cause damage to the pancreas [16]. During these pathological conditions, qPSCs become activated where they undergo morphological and functional changes as they transition from a quiescent phenotype to an active one. aPCSs contribute to the population of CAFs. aPSCs exhibit enhanced proliferative and migratory phenotypes when compared to qPSCs [14]. aPSCs lose their vitamin A droplets, display altered morphological features including ER expansion and upregulate expression of various markers including vimentin, fibroblast activation protein-α (FAP-α) and α-smooth muscle actin (αSMA). aPSCs perform large scale production of extracellular matrix (ECM) proteins and extracellular signalling factors [17,18]. Collagens, fibronectin, laminin, matrix metalloproteases (MMPs) and tissue inhibitors of metalloproteases (TIMPs) are also produced in significant quantities by aPSCs.

## 4. Interactions between Pancreatic Cancer Cells and Pancreatic Stellate Cells

While the primary function of aPSCs in PDAC tumorigenesis is to produce ECM components and form the physical desmoplasia, there is growing recognition of the important role of PSC–tumour cell interactions in promoting all aspects of PDAC pathogenesis. There is compelling evidence for a dynamic cross-talk between PSCs and cancer cells [13,19], which contribute not only to the TME but also to tumour progression, survival, metastasis and chemoresistance mechanisms in PDAC [20,21,22,23].

Most solid tumours exhibit altered metabolic pathways in the form of the Warburg or reverse Warburg effects. However, this is often insufficient for tumour growth, and further metabolic reprogramming is required. It has been shown that PSC paracrine signalling increases pancreatic cancer cell proliferation and can regulate cancer cell metabolism, thereby aiding tumorigenesis [24,25]. Paracrine signalling from cancer cells to PSCs and vice versa aids in PDAC pathogenesis. For example, an unknown signal from pancreatic tumour cells induces autophagy in recipient PSCs which leads to enhanced alanine production. The tumour cells internalise this alanine and use it as an alternative carbon source for the TCA-cycle after its conversion to pyruvate [26]. Autophagy plays a role in PSC activation, suggesting it may represent a productive area of research in the future [27]. Interestingly, this paracrine alteration of tumour metabolism is believed to occur in tandem with genetic mutations, such as mutations in KRAS which also acts to rewire PDAC metabolic pathways [28].

Most PDAC tumours develop from malignant lesions termed pancreatic intraepithelial neoplasias (PanINs) [29]. It has been shown that populations of aPSCs surround these lesions and, through secretory outputs, promote tumour progression from this precursor stage into invasive PDAC [30]. In an orthotopic model, athymic mice injected with PDAC cell lines alone or in combination with aPSCs, it was observed that primary tumour size, metastatic growth and levels of fibrosis within the tumours were significantly higher in PSC-bearing tumours [31]. Interestingly, this study also found that metastatic tumours stained positively for α-SMA and suggested that PSCs may travel with cancer cells to invade new sites. PSCs also contribute to chemoresistance observed in patients undergoing gemcitabine treatment by sequestering the drug and thus limiting its effectiveness on cancer cells [32]. Another study identified that fibronectin released by human PDAC-derived PSCs could reduce the effects of gemcitabine on cancer cells [33].

Proteomic profiling of aPSCs revealed there were 641 proteins secreted by these cells. The same profiling revealed 46 proteins that were unique to the secretome of qPSCs [34]. Amongst these proteins, aPSC release large quantities of interleukins (ILs) including IL-1, IL-6, IL-8 and IL-10; vascular endothelial growth factor (VEGF); platelet-derived growth factor (PDGF); fibroblast growth factor (FGF); and stromal derived factor 1 (SDF1α) (also called CXCL12) [35]. Many of these are important mediators of PSC and PDAC cell communication [36]. One of the most interesting chemokines in PDAC-PSC interactions is SDF-1α. It is involved in the progression of multiple disease types including cancer [37]. SDF-1α signalling can regulate tumour cell migration, proliferation, angiogenesis, EMT status and metastasis [38]. Interestingly, CXCR4, the primary SDF-1α receptor, is overexpressed in pancreatic cancer, and multiple studies have shown that SDF-1α regulates the proliferation, invasion, migration and metastasis of PDAC cells [39,40]. Additionally, SDF-1α is secreted from PDAC-derived PSCs in large quantities and is capable of modulating PDAC tumorigenesis [41]. SDF-1α also enhances PDAC chemoresistance to gemcitabine [42], while conditioned media isolated from PSCs enhance PDAC cell invasion [41] and proliferation [24].

Regulation of PSC activation and pro-tumorigenic processes associated with PSC activation is currently a relatively unexplored avenue of therapeutic interrogation in PDAC. It is tempting to suggest that targeting these cells may provide a substantial benefit to PDAC patients as a way of suppressing oncogenic signalling in the TME. Given that the UPR is known to play an important role in regulation of secretion, and the impact that this regulation can have on multiple cell processes, understanding its role in PSC phenotype may offer novel insight into PDAC progression.

## 5. The Endoplasmic Reticulum and ER Stress

The ER is an important organelle with key roles in proteostasis, lipid metabolism and drug detoxification. Perturbation of ER homeostasis results in a condition termed “ER stress” that is characterized by a build-up of unfolded proteins in the ER lumen [43]. This accumulation of unfolded and misfolded proteins in the ER is in large part due to an imbalance between protein folding demand and protein folding capacity of the ER [44]. In cancer, rapid cell proliferation and the consequent high rates of protein synthesis can lead to ER stress. Furthermore, localised depletion of oxygen, nutrients and glucose in the tumour microenvironment can also give rise to ER stress. The cellular response to ER stress is the activation of the unfolded protein response (UPR), a stress response signalling pathway that works to bring about restoration of ER homeostasis or, if this is not possible, cell death [45].

## 6. The Unfolded Protein Response

**Figure 1:**
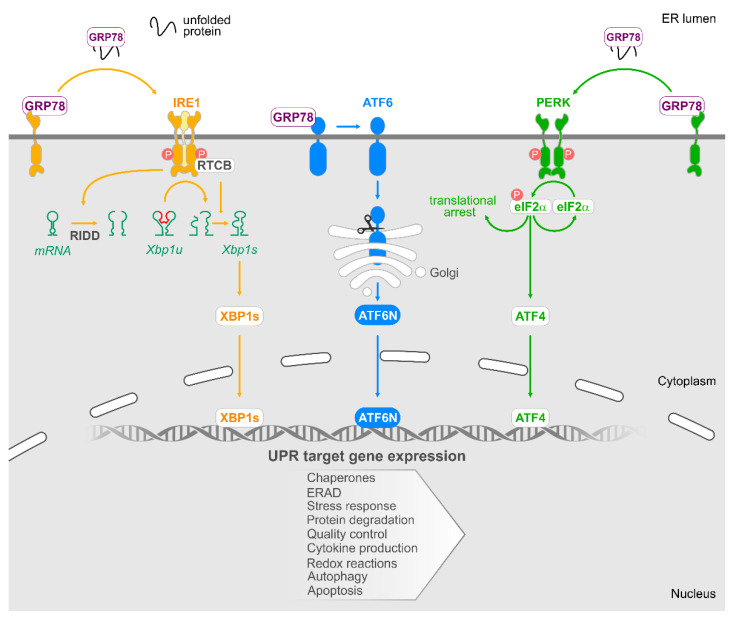
The unfolded protein response. Accumulation of unfolded proteins within the ER lumen leads to the dissociation of GRP78 from three ER stress sensors IRE1, PERK and ATF6, and their subsequent activation.Activation of IRE1 results in the removal of a 26 nucleotide intron from XBP1u mRNA. RTCB acts to ligate the mRNA, and this product is translated into the transcription factor XBP1s. Additionally, activated IRE promotes regulated IRE1 dependent decay (RIDD). RIDD activity of IRE1 is responsible for the cleavage of a subset of RNAs/miRNAs. When ATF6 is activated, it is cleaved in the Golgi to form an active transcription factor ATF6N. Upon activation, PERK phosphorylates eIF2α, and this results in the attenuation of global translation. However, the transcription factor ATF4 is active, and it acts to upregulate a subset of genes. The transcriptional programme mediated by XBP1s, ATF4 and ATF6N promotes expression of chaperones, components of ER associated protein degradation (ERAD), increased ER capacity and reduced protein translation to ameliorate ER stress and restore homeostasis. Additionally, UPR-mediated gene expression also directly impact autophagy, cytokine production and apoptosis.

The UPR is controlled by three ER-anchored receptors, namely, inositol-requiring enzyme 1 (IRE1), protein kinase RNA-like ER kinase (PERK) and activating transcription factor 6 (ATF6). Glucose-related protein 78 (GRP78, also known as binding immunoglobulin protein (BiP)), an ER chaperone protein present in the ER lumen, blocks activation of IRE1, PERK and ATF6 by binding to their ER luminal domains. Build-up of misfolded proteins in the ER lumen, which occurs under ER stress conditions, promotes GRP78 dissociation resulting in the activation of IRE1, PERK and ATF6 and their downstream signalling pathways as described in Figure 1.

### 6.1. IRE1

Following dissociation of GRP78, IRE1 oligomerises and trans-autophoshorylates enabling its RNase activity [46,47]. The IRE1 RNase domain has two key functions, processing of X Box Binding protein 1 (XBP1) mRNA and the regulated IRE1-dependent decay (RIDD). The processing of XBP1 mRNA is the most well understood output of IRE1 RNase activity. IRE1 removes a 26 nucleotide intron from XBP1 mRNA, and its re-ligation by RNA 2′,3′-cyclic phosphate and 5′-OH ligase (RTCB) produces an mRNA commonly referred to as spliced XBP1 (*XBP1s*) [48]. When translated, this produces a potent transcription factor, XBP1s, which upregulates genes involved in relieving ER stress (ER chaperones, components of the ER-associated degradation machinery) [49]. Our understanding of RIDD activity of IRE1 is limited. RIDD activity is responsible for the cleavage of a subset of RNAs, including mRNAs and miRNAs, many of which remain to be identified [50]. The IRE1′s RIDD activity may represent a mechanism to reduce pressure on the ER by slowing the influx of newly synthesised proteins [51]. In addition to RNase activity, the kinase domain of IRE1 has been linked to c-Jun N terminal kinase (JNK) signalling via recruitment of TRAF2 [52].

### 6.2. PERK

Upon GRP78 dissociation, PERK dimerises, undergoes trans-autophosphorylation of its cytosolic domain and phosphorylates its primary substrate, eukaryotic translation initiation factor 2A (eIF2α). This leads to attenuation of global mRNA translation thus reducing the load of nascent proteins entering the ER [53]. In contrast to the general stall of mRNA translation, select genes, with more than one open upstream reading frame (uORF) or internal ribosome entry sequence (IRES), are paradoxically upregulated [54,55,56]. The best understood example of this is activating transcription factor 4 (ATF4), which leads to increased expression of a variety of genes. These include the phosphatase growth arrest and DNA-damage inducing protein (GADD34) which promotes the dephosphorylation of eIF2α and thus restoration of mRNA translation [57] and C/EBP homologous protein (CHOP), a transcription factor reported to influence cell fate by regulating expression of pro-apoptotic BCL-2 family members [58].

### 6.3. ATF6

Release of GRP78 causes a conformational change in ATF6 revealing two Golgi localisation signals [59]. Upon translocation to the Golgi, ATF6 is cleaved by S1 and S2 proteases to form an active transcription factor referred to ATF6N [60]. ATF6N upregulates expression of genes with an ER stress response element (ERSE) within their promoter, including ER chaperones such as GRP78 [61]. In addition to upregulating ER chaperone expression, ATF6N also feeds into the IRE1 pathway by inducing expression of *XBP1* mRNA [62].

## 7. UPR Activation in PDAC

**Figure 2:**
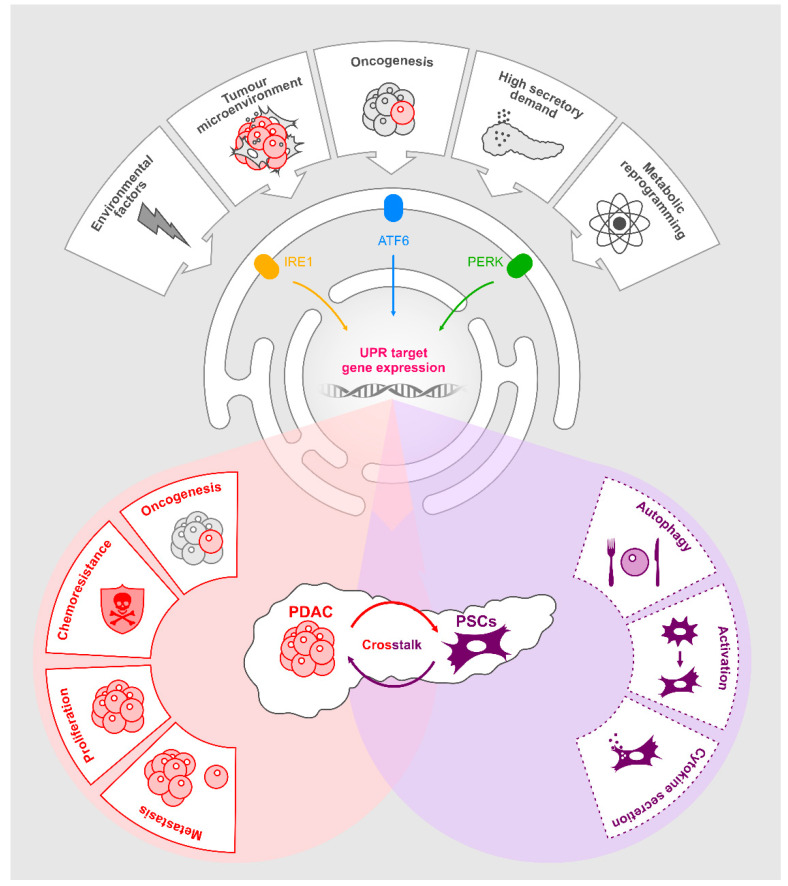
The Role of unfolded protein response (UPR) in pancreatic ductal adenocarcinoma (PDAC).Various stresses including environmental factors, the tumour microenvironment, oncogenesis, high secretory demand and metabolic reprogramming activate the UPR in pancreatic cancer. This results in upregulation of UPR gene targets. Many of these gene promote oncogenic pathways in PDAC cells. UPR activation promotes a pro-tumorigenic pancreatic stellate cell (PSCs) phenotype. Paracrine signalling from PDAC cells and PSCs, as a consequence of UPR activation in PDAC, also promotes tumorigenic behaviours.

PDAC tumours and the cells comprising their surrounding microenvironments are reported to exhibit basal ER stress due to their hypoxic, nutrient-deprived state [63]. Thus, it is likely that PDAC tumours possess a basally active UPR profile. This adaptive UPR may contribute to tumorigenesis and progression. In Figure 2, we will examine the evidence supporting a role for the UPR in PDAC and the opportunities this may create for therapeutic intervention.

## 8. GRP78

The most commonly researched UPR component in pancreatic cancer is GRP78. It is important to point out that GRP78 expression is not itself a marker of UPR activity due to its constitutive expression in normal cells. However, elevated levels of GRP78 are commonly observed under conditions of ER stress, and its expression is increased upon activation of the UPR. Increased expression of GRP78 has been correlated to poor disease prognosis and progression of multiple cancer types including prostate cancer [64], ovarian cancer [65] and B-cell lymphoma [66]. Similar to what is observed in other cancer types, increased GRP78 expression is evident in PDAC tissue and has been reported to correlate with a poor prognosis for PDAC patients [67,68].

Elevated expression of GRP78 is a feature of PDAC cells when compared to normal pancreatic ductal cells [69], although how increased levels of GRP78 contribute to PDAC progression remains to be clearly defined. Transcriptomic analysis of the PDAC cell line S2-VP10, with or without shRNA-mediated knockdown of *GRP78*, suggests its influence may be broad-reaching with changes noted in key signalling pathways including cell-cycle, apoptosis and actin-cytoskeleton regulation [70]. PDAC cell lines containing shGRP78 displayed reduced migratory, invasive and clonogenic properties compared to their shSCR (scrambled) counterparts [70]. In the same study, lower GRP78 expression limits the tumour initiation potential of S2-VP10 cells using subcutaneous limiting dilution xenograft assays. Of the tumours that did form, they exhibited a higher percentage of apoptotic cells and a reduced tumour volume [70].

A role for GRP78 in murine models of PDAC has also been observed. In a *Pdx1-Cre; Kras^G12D/+^; p53^f/+^* (PKC) mouse model of PDAC, high GRP78 was observed in the pancreata of 2 month old mice that had developed tumours by 3 months old. Interestingly, in the same genetic background, mice pancreata bearing an additional *Grp78^f/+^* allele exhibited reduced pancreatic tumourigenesis [71].

GRP78 can also impact chemoresistance, and some have suggested that a combination therapy that includes an anti-GRP78 agent may be beneficial in the treatment of pancreatic cancer [72,73]. One study demonstrated that silencing *GRP78* reduces efflux by ATP-binding cassette transporters [74]. In this study, cells treated with siGRP78 in combination with chemotherapeutic drugs exhibited significantly more cell death than in siSCR counterparts. The study demonstrated that in vitro, in PDAC cell lines, gemcitabine treatment increased GRP78 levels. In addition, low levels of GRP78 increased sensitivity to chemotherapy in vitro. These results suggested that high GRP78 levels could contribute to the poor patient response to chemotherapy [74].

Despite these studies, there remains significant questions regarding the precise role of GRP78 in PDAC. High levels of GRP78 are present in PDAC, and these studies indicate that reducing GRP78 has anti-tumour effects. However, these observations seem paradoxical when UPR activation in the context of altered GRP78 expression is taken into account. While it is likely that the levels of GRP78 reflect perturbations in proteostasis in PDAC, there remains many unknowns regarding the precise role of this protein in PDAC as well as other cancers. Precisely why GRP78 is increased in PDAC has not been extensively explored. High levels of expression could simply reflect enhanced ER homeostasis that is common in many cancer cells. Alternatively, high GRP78 could be a consequence of rapid proliferation of the cells, which would lead to high levels of protein synthesis. There remains much to learn regarding the molecular mechanisms governing its expression and regulation in PDAC.

## 9. IRE1, PERK and ATF6

While IRE1 [51,75] and PERK [76,77] have well-documented functions in pancreatic cells relating to insulin biosynthesis and glucose homeostasis, there is limited knowledge pertaining to their role in the progression and spread of PDAC.

Similar to other cancer types (triple negative breast cancer, prostate cancer and colon cancer), a role for IRE1-dependent signaling in maintaining proliferative capacity of PDAC cells has been reported [78,79,80]. Suppression of IRE1 RNase activity (using STF-083010, 2-hydroxy-1-naphthaldehyde, 3-ethoxy-5,6-dibromosalicylaldehyde, toyocamycin) in a panel of 14 PDAC cell lines is associated with a decreased proliferative capacity [81]. Induction of anterior gradient protein 2 homolog (AGR2) by the IRE1 and ATF6 arms of the UPR may contribute to the enhanced proliferation [82]. Its expression has also been reported to increase in early PDAC neoplasias [83]. The elevated expression of this member of the ER-associated protein disulphide isomerase family may enhance the protein folding capacity of the ER, thus helping cells maintain the increased proliferation characteristic of the initiating stages of PDAC development [83]. In another study, STF-083010 reduced the viability of PDAC cells. In combination with chloroquine, a well-known autophagy inhibitor, STF-083010 had an additive inhibitory effect on PDAC growth [68]. PERK activation has been linked to increased tumorigenesis. In a study utilizing a PERK inhibitor, the authors demonstrated that PERK inhibition increased apoptosis in PDAC cells and reduced tumour burden when used in vivo in PDAC xenografts [84]. Interestingly, a recent study linked PERK activity to pancreatic tumour metastasis to the liver, a frequent organ for PDAC metastasis. Single PDAC cells in the liver exhibited selective activation of the PERK arm of UPR without IRE1 activation [85]. The authors showed that this enabled PDAC cells to evade T cell killing and remain ”dormant” in the liver. In mouse models recapitulating PDAC tumour cell latency, inducible expression of XBP1s in latent cancer cells in combination with T cell depletion enabled these cancer cells to proliferate again, and metastasis ensued [85]. Taken together, these studies suggest that both the IRE1 and PERK arms of the UPR are active in PDAC cells; however, there remain many unknowns regarding the extent of their activation and their specific roles in promoting PDAC progression.

As well as ductal cells, enzyme producing acinar cells have also been suggested as PDAC cells of origin [86]. Acinar cells have an extensive ER network owing to their high rates of protein synthesis and processing, making them particularly susceptible to ER stress and UPR activation. Genetic ablation of UPR components in acinar cells has demonstrated an important role for the UPR in cell health and normal function. Namely, in conditional acinar specific XBP1^fl/fl^ mice, XBP1 loss resulted in apoptosis of acinar cells demonstrating the importance of this gene in acinar cells [87]. PERK was also shown to be vital for acinar cell survival in mouse acinar cells [77]. Taking these features into account, it is tempting to hypothesize that acinar cells may utilize XBP1 and PERK to promote cell survival in PDAC.

## 10. UPR in PDAC Chemoresistance

Limited efficacy and acquired resistance to chemotherapy are significant issues in treatment of PDAC. Gemcitabine resistance is commonly reported and has led to a surge in research for combinatory treatments that can overcome chemoresistance. While gemcitabine can be an effective chemotherapeutic, it is also thought to contribute to relapse of a more aggressive tumour type. Gemcitabine is known to induce PDAC epithelial to mesenchymal transition, enhance survival of cells that persist after treatment [88,89,90], increase inflammatory cytokine signalling [91] and promote pro-tumorigenic macrophage infiltration [92].

A possible mechanism of chemoresistance may utilise pro-survival signalling of the UPR such that cancer cells can evade chemotherapy-induced apoptosis. It was suggested that a cancer stem cell-like population isolated from a PANC1 cell line displayed divergent UPR responses after treatment with gemcitabine, where phospho-PERK and ATF6N were increased, and phospho-IRE1 was decreased [93]. Interestingly, a similar divergence of UPR response has also been reported in prostate cancer cells in response to androgen treatment, suggesting this separation of the canonical UPR arms may not be an uncommon phenomenon [79]. Another study identified synergistic effects between gemcitabine and inhibitors of fatty acid synthesis that lead to decreases in stemness and increased ER-stress-associated apoptosis in PDAC cells [94].

## 11. UPR Activation in PSCs

As previously discussed, PSCs are a key cell type within the pancreas, and growing evidence suggests that they promote PDAC progression. PSCs may also impact responsiveness of PDAC to chemotherapy [33]. The role of UPR signalling in PSCs has not been extensively explored, although mitochondrial impairment [95] and fatty acid exposure [96] have both been reported to induce UPR activity in PSCs. However, the expression of UPR-related genes prior to and during the activation process in PCSs is unknown.

Although knowledge of UPR activation in PSCs is lacking, it is informative to consider the role of the UPR in HSCs. PSCs share many functional and phenotypical characteristics with HSCs. Transcriptional analysis reveals that HSCs and PSCs share very similar genetic profiles [97]. In HSCs, IRE1 signalling has been shown to act upstream of autophagy leading to their activation [98,99]. This activity is exacerbated by treatment with the ER stress-inducing agent, brefeldin A [100]. Recently, a further connection between HSC activation and UPR was suggested, where co-culture of HSC and hepatic carcinoma cells resulted in ER stress in stellate cells. HSCs were activated, and this could be prevented by used of an IRE1 inhibitor. This suggests a role for IRE1 activity in the activation of HSCs. The role for IRE1 in the activation of PSCs remains to be elucidated. If the UPR plays important roles in both PDAC and PSC cells, it could prove to be a pivotal control point in the interactions between these two cell types.

## 12. UPR-Mediated Regulation of Cytokines and Implications for PDAC

The unique PDAC microenvironment promotes tumour development and is a major source of cytokines that promote oncogenesis. It has been hypothesized that the UPR represents a potential crossroads between ER stress and inflammatory signalling [101]. UPR can activate gene expression of cytokines both directly (via XBP1) and indirectly (via NF-_K_B activation for example). This may be an important consideration in the context of pancreatic cancer.

The role and mechanics of UPR-mediated cytokine regulation in inflammatory and disease conditions is extensively reviewed elsewhere [101]. A recent paper by Logue et al. [78] reported that the common chemotherapeutic agent paclitaxel enhanced levels of cytokines such as IL-8 and IL-6 in an IRE1-associated manner in triple negative breast cancer cells, and they were reduced upon IRE1 RNase inhibition [78]. These cytokines are important regulators of PDAC tumours. For example, IL-6 plays important roles in PDAC including regulation of migration [102], disease progression [103] and chemoresistance [104]. IL-8 is also an important player in PDAC with roles in cancer cell “stemness” [105], angiogenesis [106] and metastasis [107]. While most evidence of UPR-regulated cytokines in cancer involves the IRE1 signalling axis, both PERK [108,109] and ATF6 [110] also regulate inflammatory pathways. If PDAC and stellate cells have basally active UPR and if expression of cytokines are regulated by IRE1 (or other UPR arms), the UPR may represent an important crossroads in the regulation of communications between PDAC tumour cells and their stromal counterparts.

The concept of transmissible ER stress (TERS) is quickly gaining traction in the UPR field. This concept is based on the observation that cells experiencing ER stress secrete some unknown factor(s) capable of inducing ER-stress response/UPR in the nearby recipient cells. Originally identified in myeloid cells [111], TERS has also been reported in tumour cells [112] and cells of the central nervous system (CNS) [113]. The idea of TERS contributing to tumorigenesis is intriguing, whereby cells experiencing ER stress stimulate a cascade that enhances the UPR signalling of neighbouring cells. The signals that make up TERS remain to be identified, but given the evidence that IRE1 regulates cytokine signalling and metabolic pathways [114,115], TERS represents a strong candidate for further investigation and may have relevance to pancreatic cancer.

## 13. Concluding Remarks

Pancreatic cancer is predicted to rise from the fourth to second highest mortality rate for cancer-related deaths in developed countries in the next decade [1]. There is a vital need to investigate PDAC, with a focus on developing effective therapies that improve patient outcome. Pancreatic tumours, like most other solid tumours, have high basal levels of UPR, possibly due to a combination of intrinsic factors (e.g., oncogenesis and metabolic changes) or extrinsic microenvironmental factors (e.g., hypoxia, nutrient deprivation). The UPR has emerged not only a mechanism to alleviate ER stress but also as a potent regulator of secreted factors such as cytokines that impact cancer progression [78,116].

Targeting UPR in PDAC may be highly beneficial. Although the UPR is considered to be an integrated pathway requiring coordinated activation of IRE1, PERK and ATF6, independent activation of these stress sensors under certain conditions highlights the complexity of the pathway [79,93]. Development of selective UPR inhibitors enables direct targeting of perturbed arms of the pathway in isolation from the others [117]. For example, STF-083010, MKC8866 and B-109 all act to inhibit the RNase activity of IRE1, while small molecules such as KIRA6 are kinase binders. Small molecule inhibitors to target PERK, such as AMG44, have also been developed. Although some of these molecules have been tested in cancer, it will be of interest to examine their impact in a PDAC setting. Given the failings of modern chemotherapy against PDAC and the likelihood that combination treatments will be required to provide an effective anti-tumorigenic response in patients, the emergence of highly specific, selective inhibitors against UPR components may represent a viable therapeutic strategy.

A key aspect of PDAC biology lies in its unique TME. In this setting, UPR activation likely affects both autocrine and paracrine interactions, and UPR targeted therapies could offer a novel approach to modulate these PDAC signals. As the dense stroma surrounding pancreatic tumours creates a physical barrier against current treatments (largely chemotherapeutics), targeting the interactions that foster the expansion and growth of this barrier will enhance the efficiency of already available treatments. If the UPR does in fact regulate pancreatic cytokines and growth factors, promoting a tumour microenvironment that supports PDAC progression, it represents an exciting prospect for future studies in the field of pancreatic cancer research.
